# 2,4-Bis(4-fluoro­phen­yl)-2,3-dihydro-1*H*-1,5-benzodiazepine

**DOI:** 10.1107/S1600536811015455

**Published:** 2011-04-29

**Authors:** Zeliha Baktır, Mehmet Akkurt, S. Samshuddin, B. Narayana, H. S. Yathirajan

**Affiliations:** a Department of Physics, Faculty of Sciences, Erciyes University, 38039 Kayseri, Turkey; b Department of Studies in Chemistry, Mangalore University, Mangalagangotri 574 199, India; c Department of Studies in Chemistry, University of Mysore, Manasagangotri, Mysore 570 006, India

## Abstract

In the title compound, C_21_H_16_F_2_N_2_, the seven-membered 1,4-diazepine ring of the benzodiazepine ring system adopts a distorted-boat conformation. The benzene ring of this system makes dihedral angles of 18.6 (2) and 78.8 (2)° with those of two fluoro­phenyl substituents. In the crystal, inversion dimers linked by two weak C—H⋯F hydrogen bonds generate *R*_2_^2^(20) ring motifs. There are also weak N—H⋯π and C—H⋯π inter­actions.

## Related literature

For related structures, see: An *et al.* (2007 ▶); Bibila Mayaya Bisseyou *et al.* (2010 ▶); Harrison *et al.* (2005 ▶); Peeters *et al.* (1997 ▶). For puckering parameters, see: Cremer & Pople (1975 ▶). For graph-set nomenclature of hydrogen bonds, see: Bernstein *et al.* (1995 ▶).
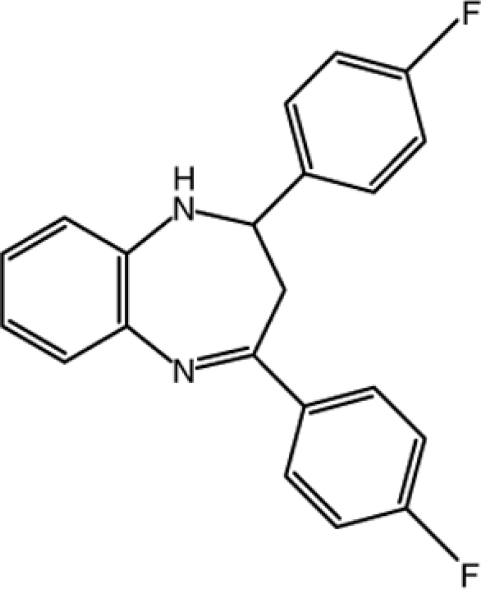


## Experimental

### 

#### Crystal data

C_21_H_16_F_2_N_2_
*M* *_r_* = 334.36
Monoclinic, 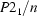

*a* = 12.9151 (4) Å
*b* = 6.0438 (3) Å
*c* = 21.2851 (7) Å
β = 92.147 (3)°
*V* = 1660.27 (11) Å^3^
*Z* = 4
Mo *K*α radiation
μ = 0.10 mm^−1^
*T* = 294 K
0.20 × 0.20 × 0.20 mm

#### Data collection

Rigaku R-AXIS RAPID-S diffractometer
Absorption correction: refined from Δ*F* (*XABS2*; Parkin *et al.*, 1995 ▶) *T*_min_ = 0.981, *T*_max_ = 0.981
3413 measured reflections
3413 independent reflections
1226 reflections with *I* > 2σ(*I*)

#### Refinement

*R*[*F* ^2^ > 2σ(*F* ^2^)] = 0.061
*wR*(*F* ^2^) = 0.151
*S* = 1.04
3413 reflections
233 parameters
2 restraints
H atoms treated by a mixture of independent and constrained refinement
Δρ_max_ = 0.16 e Å^−3^
Δρ_min_ = −0.18 e Å^−3^

### 

Data collection: *CrystalClear* (Rigaku/MSC, 2005 ▶); cell refinement: *CrystalClear*; data reduction: *CrystalClear*; program(s) used to solve structure: *SIR97* (Altomare *et al.*, 1999 ▶); program(s) used to refine structure: *SHELXL97* (Sheldrick, 2008 ▶); molecular graphics: *ORTEP-3 for Windows* (Farrugia, 1997 ▶); software used to prepare material for publication: *WinGX* (Farrugia, 1999 ▶).

## Supplementary Material

Crystal structure: contains datablocks global, I. DOI: 10.1107/S1600536811015455/hb5848sup1.cif

Structure factors: contains datablocks I. DOI: 10.1107/S1600536811015455/hb5848Isup2.hkl

Supplementary material file. DOI: 10.1107/S1600536811015455/hb5848Isup3.cml

Additional supplementary materials:  crystallographic information; 3D view; checkCIF report

## Figures and Tables

**Table 1:** Hydrogen-bond geometry (Å, °) *Cg*1 and *Cg*2 are the centroids of the benzene rings of the two fluorophenyl substituents (C10–C15 and C16–C21, respectively).

*D*—H⋯*A*	*D*—H	H⋯*A*	*D*⋯*A*	*D*—H⋯*A*
C5—H5⋯F1^i^	0.93	2.54	3.469 (6)	175
N1—H1*N*⋯*Cg*2^i^	0.86 (3)	2.82 (5)	3.601 (4)	151 (4)
C2—H2⋯*Cg*1^ii^	0.93	2.89	3.640 (5)	138
C11—H11⋯*Cg*2	0.93	2.79	3.494 (5)	134

Symmetry codes: (i) 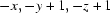; (ii) 
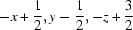
.

**Fig. 1.**
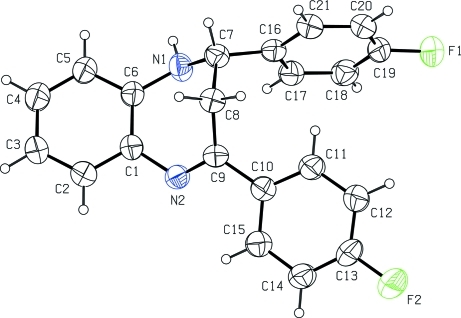
View of the structure of (I) with displacement ellipsoids for non-H atoms drawn at the 30% probability level.

**Fig. 2.**
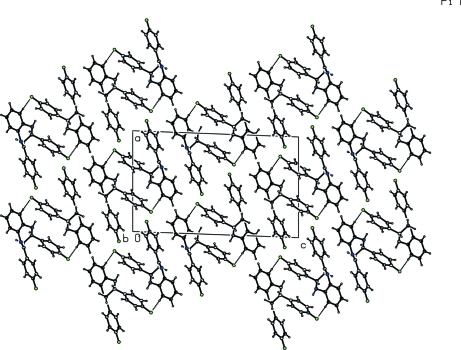
Packing diagram of the title compound viewed down the b axis. Hydrogen bonds are shown as dotted lines.

## supplementary crystallographic information

### Comment

The crystal structures of some 1,5-benzodiazepines, *viz*., 2-[2-(4-methoxyphenyl)-2,3-dihydro-1*H*-1,5-benzodiazepin-4-yl]phenol (Bibila Mayaya Bisseyou *et al.*, 2010), 1-(2-bromo-5-methoxyphenyl)-8-chloro-6-(2-fluorophenyl)-4*H*-1,2,4-triazolo[4,3-*a*][1,4] benzodiazepine (Harrison *et al.*, 2005), 5-(4-fluorophenyl)-1,8-dimethyl-2-(*p*-toluoylaminomethyl)-2,3-dihydro-1*H*-1,4-benzodiazepine monohydrate (Peeters *et al.*, 1997) and 2,4-bis(4-chlorophenyl)-2-methyl-2,3-dihydro-1*H*-1,5-benzodiazepine (An *et al.*, 2007) have been reported. In continuation of this work, the title compound, (I), is synthesized and its crystal structure is reported here.

The seven-membered 1,4-diazepine ring (C1/C6–C9/N1/N2) of the benzodiazepine ring system (C1–C9/N1/N2) adopts a distorted-boat conformation [the puckering parameters (Cremer & Pople, 1975) for this eleven-membered ring system are: Q_2_ = 0.917 (4) Å, Q_3_ = 0.155 (4) Å, φ_2_ = 16.6 (3)° and φ_3_ = 92.6 (17)°] as shown in Fig. 1. The benzene ring (C1–C6) of this system forms dihedral angles of 18.6 (2)° and 78.8 (2)° with the benzene rings (C10–C15 and C16–C21) of two fluorophenyl fragments, respectively which make a dihedral angle of 62.1 (2)° with each other.

In the crystal, the two weak C—H···F hydrogen bonds link pairs of inversion-related molecules to form cyclic centrosymmetric dimers containing the *R*^2^_2_(20) ring motif (Bernstein *et al.*, 1995; Table 1, Fig. 2). In addtion, three C—H···π interactions are observed (Table 1).

### Experimental

To a solution of 4,4'-difluoro chalcone (2.44 g, 0.01 mol) in ethanol (30 ml) a few drops of piperidine and 1, 2-diaminobenzene (1.08 g, 01 mol) were added. The mixture was heated under reflux for 10 h. The reaction mixture was cooled and poured into 50 ml ice-cold water. The precipitate was collected by filtration and purified by recrystallization from ethanol. Pale yellow blocks of (I) were grown from DMF by slow evaporation method in 66% yield (m. p.: 409 K).

### Refinement

The amine and methine H atoms were placed from a Fourier map and positional parameters were constrained to ride on their parent atom by applying the N–H and C–H *DFIX* restraints of 0.86 (1) and 0.98 (1) Å, respectively. Their isotropic displacement parameters were set to be 1.2U_eq_ of the carrier atoms. The other H atoms were positioned geometrically [C—H = 0.93 and 0.97Å for aromatic and methylene H atoms, respectively] and allowed to ride on their parent C atoms, with *U*_iso_(H) = 1.2*U*_eq_(C). Owing to the large number of weak high-angle reflections, the ratio of observed to unique reflections is low (36%).

### Crystal data

**Table:**

C_21_H_16_F_2_N_2_	*F*(000) = 696
*M**_r_* = 334.36	*D*_x_ = 1.338 Mg m^−^^3^
Monoclinic, *P*2_1_/*n*	Mo *K*α radiation, λ = 0.71073 Å
Hall symbol: -P 2yn	Cell parameters from 1691 reflections
*a* = 12.9151 (4) Å	θ = 2.5–26.3°
*b* = 6.0438 (3) Å	µ = 0.10 mm^−^^1^
*c* = 21.2851 (7) Å	*T* = 294 K
β = 92.147 (3)°	Block, pale yellow
*V* = 1660.27 (11) Å^3^	0.20 × 0.20 × 0.20 mm
*Z* = 4	

### Data collection

**Table:**

Rigaku R-AXIS RAPID-S diffractometer	3413 independent reflections
Radiation source: Sealed Tube	1226 reflections with *I* > 2σ(*I*)
Graphite Monochromator	*R*_int_ = 0.000
Detector resolution: 10.0000 pixels mm^-1^	θ_max_ = 26.5°, θ_min_ = 3.2°
dtprofit.ref scans	*h* = −16→16
Absorption correction: part of the refinement model (Δ*F*) [*XABS2* (Parkin *et al.*, 1995); Cubic fit to sinθ/λ, 24 parameters]	*k* = 0→7
*T*_min_ = 0.981, *T*_max_ = 0.981	*l* = 0→26
3413 measured reflections	

### Refinement

**Table:**

Refinement on *F*^2^	Primary atom site location: structure-invariant direct methods
Least-squares matrix: full	Secondary atom site location: difference Fourier map
*R*[*F*^2^ > 2σ(*F*^2^)] = 0.061	Hydrogen site location: inferred from neighbouring sites
*wR*(*F*^2^) = 0.151	H atoms treated by a mixture of independent and constrained refinement
*S* = 1.04	*w* = 1/[σ^2^(*F*_o_^2^) + (0.*P*)^2^ + 1.2828*P*] where *P* = (*F*_o_^2^ + 2*F*_c_^2^)/3
3413 reflections	(Δ/σ)_max_ < 0.001
233 parameters	Δρ_max_ = 0.16 e Å^−^^3^
2 restraints	Δρ_min_ = −0.18 e Å^−^^3^

### Special details

**Table:**

Geometry. Bond distances, angles *etc*. have been calculated using the rounded fractional coordinates. All su's are estimated from the variances of the (full) variance-covariance matrix. The cell e.s.d.'s are taken into account in the estimation of distances, angles and torsion angles
Refinement. Refinement on *F*^2^ for ALL reflections except those flagged by the user for potential systematic errors. Weighted *R*-factors *wR* and all goodnesses of fit *S* are based on *F*^2^, conventional *R*-factors *R* are based on *F*, with *F* set to zero for negative *F*^2^. The observed criterion of *F*^2^ > σ(*F*^2^) is used only for calculating -*R*-factor-obs *etc*. and is not relevant to the choice of reflections for refinement. *R*-factors based on *F*^2^ are statistically about twice as large as those based on *F*, and *R*-factors based on ALL data will be even larger.

### Fractional atomic coordinates and isotropic or equivalent isotropic displacement parameters (Å^2^)

**Table:**

	*x*	*y*	*z*	*U*_iso_*/*U*_eq_	
F1	0.2985 (2)	0.5093 (5)	0.38988 (13)	0.1156 (16)	
F2	0.6025 (2)	−0.0519 (5)	0.58369 (17)	0.1365 (18)	
N1	−0.0014 (3)	0.2319 (7)	0.60658 (19)	0.0764 (17)	
N2	0.1477 (3)	−0.0519 (6)	0.67436 (16)	0.0667 (16)	
C1	0.0446 (4)	−0.0259 (7)	0.6923 (2)	0.0649 (17)	
C2	0.0114 (4)	−0.1609 (7)	0.7400 (2)	0.0747 (19)	
C3	−0.0881 (4)	−0.1566 (8)	0.7601 (2)	0.084 (2)	
C4	−0.1587 (4)	−0.0156 (9)	0.7299 (2)	0.090 (2)	
C5	−0.1280 (4)	0.1161 (8)	0.6815 (2)	0.083 (2)	
C6	−0.0273 (4)	0.1150 (7)	0.6613 (2)	0.0669 (17)	
C7	0.0768 (4)	0.4074 (7)	0.6097 (2)	0.0674 (17)	
C8	0.1593 (3)	0.3499 (7)	0.66113 (19)	0.0675 (17)	
C9	0.2006 (3)	0.1161 (7)	0.6576 (2)	0.0635 (17)	
C10	0.3074 (3)	0.0767 (7)	0.6375 (2)	0.0652 (17)	
C11	0.3591 (4)	0.2231 (8)	0.6009 (2)	0.080 (2)	
C12	0.4584 (4)	0.1818 (9)	0.5813 (2)	0.095 (3)	
C13	0.5044 (4)	−0.0120 (10)	0.6017 (3)	0.094 (3)	
C14	0.4580 (4)	−0.1612 (8)	0.6392 (2)	0.085 (2)	
C15	0.3579 (4)	−0.1181 (7)	0.6567 (2)	0.0735 (17)	
C16	0.1258 (3)	0.4366 (7)	0.5464 (2)	0.0628 (17)	
C17	0.1274 (3)	0.2722 (7)	0.5013 (2)	0.0710 (17)	
C18	0.1843 (4)	0.2974 (9)	0.4478 (2)	0.083 (2)	
C19	0.2394 (4)	0.4894 (10)	0.4416 (2)	0.081 (2)	
C20	0.2382 (4)	0.6561 (8)	0.4837 (2)	0.080 (2)	
C21	0.1800 (3)	0.6298 (7)	0.5363 (2)	0.0721 (17)	
H1N	−0.060 (2)	0.265 (10)	0.588 (2)	0.1640*	
H2	0.05840	−0.25840	0.75920	0.0900*	
H3	−0.10780	−0.24590	0.79310	0.1010*	
H4	−0.22690	−0.01050	0.74250	0.1080*	
H5	−0.17630	0.20920	0.66170	0.1000*	
H7	0.048 (4)	0.552 (4)	0.621 (2)	0.1640*	
H8A	0.21670	0.45220	0.65810	0.0810*	
H8B	0.12960	0.37130	0.70190	0.0810*	
H11	0.32650	0.35440	0.58870	0.0960*	
H12	0.49230	0.28040	0.55560	0.1140*	
H14	0.49250	−0.28860	0.65270	0.1020*	
H15	0.32380	−0.21960	0.68140	0.0880*	
H17	0.08980	0.14290	0.50700	0.0850*	
H18	0.18500	0.18780	0.41710	0.0990*	
H20	0.27570	0.78520	0.47760	0.0960*	
H21	0.17710	0.74420	0.56540	0.0860*	

### Atomic displacement parameters (Å^2^)

**Table:**

	*U*^11^	*U*^22^	*U*^33^	*U*^12^	*U*^13^	*U*^23^
F1	0.117 (3)	0.135 (3)	0.098 (2)	0.027 (2)	0.0457 (19)	0.034 (2)
F2	0.080 (2)	0.133 (3)	0.199 (4)	0.019 (2)	0.040 (2)	0.037 (3)
N1	0.065 (3)	0.093 (3)	0.071 (3)	−0.011 (2)	0.002 (2)	0.014 (2)
N2	0.063 (3)	0.064 (2)	0.073 (3)	0.001 (2)	0.003 (2)	0.001 (2)
C1	0.065 (3)	0.066 (3)	0.064 (3)	−0.005 (3)	0.006 (2)	0.001 (2)
C2	0.078 (4)	0.070 (3)	0.076 (3)	0.000 (3)	0.002 (3)	0.003 (3)
C3	0.088 (4)	0.088 (4)	0.077 (4)	0.001 (3)	0.020 (3)	0.014 (3)
C4	0.073 (4)	0.109 (4)	0.090 (4)	0.005 (3)	0.015 (3)	0.011 (3)
C5	0.069 (3)	0.098 (4)	0.083 (4)	0.009 (3)	0.013 (3)	0.012 (3)
C6	0.066 (3)	0.072 (3)	0.063 (3)	0.001 (3)	0.008 (2)	0.002 (2)
C7	0.069 (3)	0.069 (3)	0.064 (3)	0.004 (3)	0.000 (2)	0.004 (3)
C8	0.072 (3)	0.064 (3)	0.066 (3)	−0.001 (2)	−0.003 (2)	−0.003 (2)
C9	0.064 (3)	0.061 (3)	0.065 (3)	0.005 (2)	−0.004 (2)	0.000 (2)
C10	0.062 (3)	0.067 (3)	0.066 (3)	−0.004 (3)	−0.006 (2)	0.000 (2)
C11	0.062 (3)	0.082 (4)	0.096 (4)	0.001 (3)	0.000 (3)	0.015 (3)
C12	0.071 (4)	0.102 (4)	0.113 (5)	0.003 (3)	0.013 (3)	0.031 (3)
C13	0.056 (3)	0.105 (5)	0.123 (5)	0.012 (3)	0.015 (3)	0.010 (4)
C14	0.067 (4)	0.078 (4)	0.109 (4)	0.005 (3)	0.003 (3)	0.010 (3)
C15	0.069 (3)	0.067 (3)	0.084 (3)	−0.007 (3)	−0.002 (3)	−0.001 (3)
C16	0.061 (3)	0.061 (3)	0.066 (3)	0.004 (2)	−0.004 (2)	0.002 (2)
C17	0.073 (3)	0.068 (3)	0.072 (3)	0.003 (3)	0.003 (3)	0.001 (3)
C18	0.091 (4)	0.084 (4)	0.074 (4)	0.016 (3)	0.007 (3)	−0.006 (3)
C19	0.078 (4)	0.099 (4)	0.067 (3)	0.020 (3)	0.017 (3)	0.023 (3)
C20	0.080 (4)	0.074 (4)	0.085 (4)	0.006 (3)	0.006 (3)	0.017 (3)
C21	0.077 (3)	0.068 (3)	0.071 (3)	0.002 (3)	−0.002 (3)	0.001 (3)

### Geometric parameters (Å, °)

**Table:**

F1—C19	1.368 (5)	C14—C15	1.384 (7)
F2—C13	1.359 (6)	C16—C21	1.382 (6)
N1—C6	1.413 (6)	C16—C17	1.382 (6)
N1—C7	1.464 (6)	C17—C18	1.387 (6)
N2—C1	1.408 (6)	C18—C19	1.370 (8)
N2—C9	1.282 (6)	C19—C20	1.349 (7)
N1—H1N	0.86 (3)	C20—C21	1.381 (6)
C1—C6	1.406 (7)	C2—H2	0.9300
C1—C2	1.383 (6)	C3—H3	0.9300
C2—C3	1.370 (7)	C4—H4	0.9300
C3—C4	1.388 (7)	C5—H5	0.9300
C4—C5	1.372 (7)	C7—H7	0.98 (3)
C5—C6	1.385 (7)	C8—H8A	0.9700
C7—C8	1.539 (6)	C8—H8B	0.9700
C7—C16	1.520 (6)	C11—H11	0.9300
C8—C9	1.513 (6)	C12—H12	0.9300
C9—C10	1.479 (6)	C14—H14	0.9300
C10—C11	1.369 (6)	C15—H15	0.9300
C10—C15	1.400 (6)	C17—H17	0.9300
C11—C12	1.386 (7)	C18—H18	0.9300
C12—C13	1.376 (8)	C20—H20	0.9300
C13—C14	1.358 (8)	C21—H21	0.9300
			
C6—N1—C7	120.6 (4)	F1—C19—C18	117.4 (4)
C1—N2—C9	120.4 (4)	C18—C19—C20	123.3 (4)
C7—N1—H1N	116 (4)	C19—C20—C21	118.2 (5)
C6—N1—H1N	105 (3)	C16—C21—C20	121.2 (4)
N2—C1—C6	123.7 (4)	C1—C2—H2	119.00
C2—C1—C6	119.0 (5)	C3—C2—H2	119.00
N2—C1—C2	117.1 (4)	C2—C3—H3	121.00
C1—C2—C3	122.6 (4)	C4—C3—H3	121.00
C2—C3—C4	118.3 (4)	C3—C4—H4	120.00
C3—C4—C5	120.0 (5)	C5—C4—H4	120.00
C4—C5—C6	122.1 (5)	C4—C5—H5	119.00
C1—C6—C5	117.9 (4)	C6—C5—H5	119.00
N1—C6—C1	121.1 (4)	N1—C7—H7	113 (3)
N1—C6—C5	120.5 (4)	C8—C7—H7	107 (3)
N1—C7—C16	110.7 (4)	C16—C7—H7	107 (2)
N1—C7—C8	109.1 (3)	C7—C8—H8A	109.00
C8—C7—C16	110.9 (4)	C7—C8—H8B	109.00
C7—C8—C9	114.3 (3)	C9—C8—H8A	109.00
N2—C9—C8	122.2 (4)	C9—C8—H8B	109.00
C8—C9—C10	119.9 (4)	H8A—C8—H8B	108.00
N2—C9—C10	117.8 (4)	C10—C11—H11	119.00
C9—C10—C15	118.7 (4)	C12—C11—H11	119.00
C9—C10—C11	122.7 (4)	C11—C12—H12	122.00
C11—C10—C15	118.6 (4)	C13—C12—H12	122.00
C10—C11—C12	122.1 (4)	C13—C14—H14	121.00
C11—C12—C13	116.9 (5)	C15—C14—H14	121.00
C12—C13—C14	123.8 (5)	C10—C15—H15	120.00
F2—C13—C14	119.0 (5)	C14—C15—H15	120.00
F2—C13—C12	117.3 (5)	C16—C17—H17	119.00
C13—C14—C15	118.1 (5)	C18—C17—H17	120.00
C10—C15—C14	120.7 (4)	C17—C18—H18	121.00
C17—C16—C21	118.6 (4)	C19—C18—H18	121.00
C7—C16—C17	123.3 (4)	C19—C20—H20	121.00
C7—C16—C21	117.8 (4)	C21—C20—H20	121.00
C16—C17—C18	120.9 (4)	C16—C21—H21	119.00
C17—C18—C19	117.7 (4)	C20—C21—H21	119.00
F1—C19—C20	119.3 (5)		
			
C6—N1—C7—C8	32.4 (5)	C7—C8—C9—N2	−74.6 (5)
C6—N1—C7—C16	154.7 (4)	N2—C9—C10—C11	159.0 (4)
C7—N1—C6—C1	−67.0 (6)	N2—C9—C10—C15	−21.1 (6)
C7—N1—C6—C5	120.7 (5)	C8—C9—C10—C11	−24.2 (6)
C9—N2—C1—C6	40.9 (6)	C8—C9—C10—C15	155.7 (4)
C9—N2—C1—C2	−144.4 (4)	C9—C10—C11—C12	−178.6 (4)
C1—N2—C9—C8	5.1 (6)	C15—C10—C11—C12	1.5 (7)
C1—N2—C9—C10	−178.2 (4)	C9—C10—C15—C14	−179.7 (4)
N2—C1—C6—C5	176.5 (4)	C11—C10—C15—C14	0.2 (7)
N2—C1—C2—C3	−177.7 (4)	C10—C11—C12—C13	−1.6 (7)
C6—C1—C2—C3	−2.7 (7)	C11—C12—C13—F2	−178.8 (5)
N2—C1—C6—N1	3.9 (7)	C11—C12—C13—C14	0.1 (8)
C2—C1—C6—N1	−170.7 (4)	F2—C13—C14—C15	−179.6 (5)
C2—C1—C6—C5	1.8 (6)	C12—C13—C14—C15	1.5 (8)
C1—C2—C3—C4	2.0 (7)	C13—C14—C15—C10	−1.6 (7)
C2—C3—C4—C5	−0.5 (7)	C7—C16—C17—C18	−171.8 (4)
C3—C4—C5—C6	−0.3 (7)	C21—C16—C17—C18	1.7 (6)
C4—C5—C6—C1	−0.4 (7)	C7—C16—C21—C20	171.2 (4)
C4—C5—C6—N1	172.2 (4)	C17—C16—C21—C20	−2.6 (6)
N1—C7—C8—C9	48.6 (5)	C16—C17—C18—C19	0.6 (7)
N1—C7—C16—C21	163.2 (4)	C17—C18—C19—F1	177.5 (4)
C8—C7—C16—C17	97.9 (5)	C17—C18—C19—C20	−2.2 (8)
C8—C7—C16—C21	−75.6 (5)	F1—C19—C20—C21	−178.4 (4)
N1—C7—C16—C17	−23.3 (6)	C18—C19—C20—C21	1.3 (8)
C16—C7—C8—C9	−73.5 (4)	C19—C20—C21—C16	1.2 (7)
C7—C8—C9—C10	108.7 (4)		

### Hydrogen-bond geometry (Å, °)

**Table:**

Cg1 and Cg2 are the centroids of the benzene rings of the two fluorophenyl substituents (C10–C15 and C16–C21, respectively).

**Table:**

*D*—H···*A*	*D*—H	H···*A*	*D*···*A*	*D*—H···*A*
C5—H5···F1^i^	0.93	2.54	3.469 (6)	175
N1—H1N···Cg2^i^	0.86 (3)	2.82 (5)	3.601 (4)	151 (4)
C2—H2···Cg1^ii^	0.93	2.89	3.640 (5)	138
C11—H11···Cg2	0.93	2.79	3.494 (5)	134

Symmetry codes: (i) −*x*, −*y*+1, −*z*+1; (ii) −*x*+1/2, *y*−1/2, −*z*+3/2.
